# Survival analysis and mortality predictors of hospitalized severe burn victims in a Malaysian burns intensive care unit

**DOI:** 10.1186/s41038-018-0140-1

**Published:** 2019-01-28

**Authors:** Henry Tan Chor Lip, Jih Huei Tan, Mathew Thomas, Farrah-Hani Imran, Tuan Nur’ Azmah Tuan Mat

**Affiliations:** 1 General Surgery Department, Hospital Sultanah Aminah, Johor Bahru, Malaysia; 2 Plastic Surgery and Burns Unit, Department of Surgery, Pusat Perubatan Universiti Kebangsaan Malaysia, Cheras, Malaysia; 3 General Surgery Department, Hospital Sultan Ismail, Johor Bahru, Malaysia

**Keywords:** Burn, Degree, Inhalation injury, Mortality predictors

## Abstract

**Background:**

Prognostic measures to determine burn mortality are essential in evaluating the severity of individual burn victims. This is an important process of triaging patients with high risk of mortality that may be nursed in the acute care setting. Malaysian burn research is lacking with only one publication identified which describes the epidemiology of burn victims. Therefore, the objective of this study was to go one step further and identify the predictors of burn mortality from a Malaysian burns intensive care unit (BICU) which may be used to triage patients at higher risk of death.

**Methods:**

This is a retrospective cohort study of all admissions to Hospital Sultan Ismail’s BICU from January 2010 till October 2015. Admission criteria were in accordance with the American Burn Association guidelines, and risk factors of interest were recorded. Data was analyzed using simple logistic regression to determine significant predictors of mortality. Survival analysis with time to death event was performed using the Kaplan-Meier survival curve with log-rank test.

**Results:**

Through the 6-year period, 393 patients were admitted with a male preponderance of 73.8%. The mean age and length of stay were 35.6 (±15.72) years and 15.3 (±18.91) days. There were 48 mortalities with an overall mortality rate of 12.2%. Significant risk factors identified on simple logistic regression were total body surface area (TBSA) > 20% (*p* < 0.001), inhalation injury (*p* < 0.001) and presence of early systemic inflammatory response syndrome (SIRS) (*p* < 0.001). Survival analysis using Kaplan-Meier survival curve showed similar results with TBSA > 20%, presence of SIRS, mechanical ventilation and inhalation injury which were associated with poorer survival (*p* < 0.001).

**Conclusion:**

The predictors of mortality identified in a Malaysian BICU were TBSA > 20%, early SIRS, mechanical ventilation and inhalation injury which were associated with poorer survival outcome. The immunological response differs from individual patients and influenced by the severity of burn injury. Early SIRS on admission is an important predictor of death and may represent the severity of burn injury. Patients who required mechanical ventilation were associated with mortality and it is likely related to the severity of pulmonary insults sustained by individual patients. This data is important for outcome prognostication and mortality risk counselling in severely burned patients.

## Background

Over the past decade, improvement and advancements of health care services have allowed more severely burned patients to survive [1, 2]. Current burn management coupled with specialized intensive care treatment led to the survival of young and healthy individuals sustaining major burn injury. This would have been fatal in comparison with medical care over a century ago [2, 3]. In developed countries, burn injuries account for more than 50,000 admissions with a mortality rate of 5–6% [4, 5]. However, when complicated with inhalation injury, the death rate increases with a reported mortality rate of over 30% [5].

The purported increase in death rate is explained by the difference in characteristics of burn injuries which varies from developing and developed nations. In developed nations, the ageing individuals are the fastest growing populations especially in Europe and America [6]. In addition, the mean age of burn patients admitted for burn injury in developed nations of Canada and Spain was higher than 44.7 and 53 years old respectively [7, 8]. In comparison with a population from a developing nation of Ecuador, the injured were younger patients with a mean age of 33 years [9]. Clearly, the difference in the demography of burn population showed that younger active citizens were more prone to burn injuries in developing nations. This is a growing concern for developing nations as the younger population represents the majority of the workforce that drives the local economy.

Local data and research in predictors of burn mortality and its predictors are insufficient. In Malaysia, only one publication describing burn injury from Malaysia was identified from our literature search which was conducted in a Malaysian university hospital. The retrospective study by Chan et al. with 110 burn patients revealed that the major cause of death was due to inhalation injury and septicaemia from infected burn wounds [10]. The majority of the injured were males, and the aetiology of burn was mainly due to domestic-related injuries. Research in this area from government-owned hospitals where the majority of burn patients would be admitted due to better accessibility and affordable cost schemes for local Malaysians is not available. Therefore, this study is to investigate the risk factors of death with larger sample size and survival analysis to identify variables which may be utilized by local clinicians to determine the prognostic factors for burn death. The other objective of this study is to identify factors that may be predictors of burn mortality that may be used to triage patients into high risk for mortality in a local government-owned hospital burns unit. By identifying these predictors, we would try to understand and hence formulate treatment plans that may improve survivability in severe burn patients.

## Methods

### Patients

This is a retrospective cohort study of burn victims that were admitted to the Hospital Sultan Ismail’s burns intensive care unit (BICU). The data was retrieved from the hospital’s burns registry from January 2010 to October 2015. The hospital’s BICU is the largest burns unit in Malaysia which is managed by a specialized burns trauma surgeon, a medical officer and 17 nurses trained in burn wound. Ventilated patients were co-managed by the hospital’s intensive care unit anaesthetist.

We included all patients aged 13 or over that were admitted to the BICU from January 2010 till October 2015. Admission criteria were in accordance with the American Burn Association guidelines of partial-thickness burn of more than 20% of total body surface area (TBSA), burns involving the face, hands, feet, genitalia, perineum or major joints, inhalation injury, full-thickness burns, electrical burns, and chemical burns, and patients that do not have the social support to care for their wounds [4]. Inhalation injury is defined as the presence of a positive history of being in an enclosed burn space and clinical features which may include singed eyebrow, soot in the nostril, laryngeal edema and facial burns that suggest possible inhalation injury. TBSA estimation is performed using the Lund and Browder chart. The indication for mechanical ventilation for patients with burn inhalation injury is a complex decision based on clinical and blood parameters coupled with the clinical experience of the attending primary health caregiver. Positive clinical findings of rales, rhonchi, wheezing, tachypnea and suspicious visible supraglottic edema are the main supportive points for mechanical ventilation. Subsequent evidence of hypoxia by the arterial blood gas results further lowers the threshold for mechanical ventilation. All patients with inhalation injury received treatment in accordance with the inhalation injury treatment protocol. The protocol includes humidified oxygen to maintain oxygen saturation above 90%, chest physiotherapy, two hourly turns and positioning, aerosolize *N*-acetylcysteine with a bronchodilator, alternate aerosol 5000 units of heparin together with normal saline, regular nasotracheal suction and sputum cultures.

The BICU is a subspecialty of general surgery which receives referrals from the emergency department, local district clinics, hospitals and inter-departmental referrals for burn injury. The burn patients were managed and resuscitated initially according to the advanced trauma life support and American Burn Association guidelines. All patients with over 20% partial-thickness to full-thickness burns were started on the Parkland formula for fluid resuscitation, and inhalational protocol was initiated for all patients that had suspected inhalation injuries.

### Study design and variables

The variables chosen to predict mortality during the hospital stay included gender, age, place of injury, mechanism of injury, length of stay, partial-thickness and full-thickness TBSA burn, inhalation injury, mechanical ventilation, duration of ventilation and mechanism of burn. These variables were extracted from the burns database which is being maintained and kept by trained burns unit nurses under the direct supervision of the burns and trauma consultant surgeon. A large burn in this study is defined as patients with involvement of greater than 20% TBSA partial-thickness and full-thickness burns. All patients’ variables were recorded, and any data discrepancies were discussed prior to the final decision to enter into the burn registry.

This research was registered in accordance with the protocol with the National Medical Research Register (NMRR-15-1474-27213). Prior to the start of data collection, permission was obtained from the Malaysian Research Ethics Committee (KKM/NIHSEC/P15-1273).

Definition of systemic inflammatory response syndrome (SIRS) was in accordance with the American College of Chest Physicians (ACCP) and the Society of Critical Care Medicine (SCCM) of two or more of the following symptoms: fever of more than 38 °C or less than 36 °C, heart rate of more than 90 beats per minute, respiratory rate of more than 20 breaths per minute or arterial carbon dioxide tension (PaCO_2_) of less than 32 mmHg and abnormal white blood cell count (> 12,000/μL or < 4000/μL or > 10% immature band forms). In this current study, early SIRS were defined as patients fulfilling the SIRS criteria stated previously within the first 24 h of admission to the BICU. The duration of first 24 h was chosen as it gave adequate time to the clinical staff to assess the patient clinical and biochemical blood parameters.

### Statistical analysis

All continuous variables were expressed as mean, standard deviation (SD) or median with the corresponding first and third quartiles (Q1–Q3), and categorical variables are expressed as frequencies and percentages. Simple logistic regression was performed to evaluate the risk factors for burn-related mortality. Kaplan-Meier survival curve with the log-rank test was performed to compare the effects of the variables of interest on mortality as a time-to-death analysis. A probability value of *p* < 0.05 was considered statistically significant. The analysis was performed using SPSS for Windows version 16.0 (SPSS Inc., Chicago, USA).

## Results

### Patient demographic and comparison between survivors and non-survivors

Prospective records for patients with the ICD-10-CM T30-T32 diagnosis from January 2010 till October 2015 were screened, of which 393 patients were included. The study population had a male preponderance of 290 male patients to 103 female patients with a mean age of 35.6 (±15.72) years. Mean length of stay was 15.3 (±18.91) days. Of the 393 patients, there were 48 deaths with an overall mortality rate of 12.2%.

Malay ethnicities were the majority of burn patients with 183 (46.6%) patients, and the minority were Indians with 34 (8.7%) patients. Household and industrial workplace burns were the two commonest mechanisms that led to burn injuries with 189 and 137 patients respectively. Similarly, household and industrial burns were the two common causes of death with 21 and 16 deaths respectively. A total of 216 patients had full-thickness burns with 179 survivors and 37 mortalities. The mean duration of ventilation was 3.8 (±8.11) days. The cut-off for 100% death was seen in patients with partial-thickness and full-thickness burns of over 85.4% TBSA.

Observed mortality rates were high in patients with TBSA > 20% (85.4%), ventilated patients (97.9%), inhalation injury (77.1%) and early SIRS (79.2%) seen in Table 1. The major causes of death from burn injuries were due to infective causes (39.6%) and inhalation injury (33.3%) as shown in Table 2.

**Table 1:** Patient demographics and comparison between survivors and non-survivors in a Malaysian burns intensive care unit

Variable	All episodes, *n* (%)	Survivor, *n* (%)	Non-survivor, *n* (%)
	(*n* = 393)	(*n* = 345)	(*n* = 48)
Age^a^ (years)	35.6 (±15.7)	35.1 (±15.6)	39.7 (±15.9)
Age group (years)			
≤ 18	34 (8.7)	32 (9.3)	2 (4.2)
19–49	287 (73.0)	254 (73.6)	33 (68.8)
50–64	48 (12.2)	40 (11.6)	8 (16.7)
65–74	15 (3.8)	11 (3.2)	4 (8.3)
≥ 75	9 (2.3)	8 (2.3)	1 (2.1)
Gender			
Male	290 (73.8)	254 (73.6)	36 (75.0)
Female	103 (26.2)	91 (26.4)	12 (25.0)
Race			
Malay	183 (46.6)	165 (47.8)	18 (37.5)
Chinese	85 (21.6)	73 (21.2)	12 (25.0)
Indian	34 (8.7)	27 (7.8)	7 (14.6)
Others	91 (23.2)	80 (23.2)	11 (22.9)
LOS^a^ (days)	9 (3.0–20.0)	9 (3.0–20.0)	9 (6.0–20.3)
% TBSA^a^	18.8 (±19.5)	14.7 (±13.9)	48.7 (±26.9)
% TBSA group			
0 – 10%	160 (40.7)	156 (45.2)	4 (8.3)
10 – 20%	104 (26.5)	101 (29.3)	3 (6.3)
20 – 30%	51 (13.0)	46 (13.3)	5 (10.4)
30 – 40%	22 (5.6)	17 (4.9)	5 (10.4)
≥ 40%	56 (14.2)	25 (7.2)	31 (64.6)
Degree of burn			
Partial-thickness	342 (87.0)	306 (88.7)	36 (75.0)
Full-thickness	216 (55.0)	179 (51.9)	37 (77.1)
Inhalation injury	98 (24.9)	61 (17.7)	37 (77.1)
SIRS at presentation	169 (43.0)	131 (38.0)	38 (79.2)
Ventilation	93 (23.7)	46 (13.3)	47 (97.9)
Ventilation period^a^ (days)	10 (4.5–14.0)	10 (5.0–16.0)	10 (4.0–13.0)

*LOS* length of stay, *% TBSA* percentage total body surface area, *SIRS* systemic inflammatory response syndrome

^a^Mean ± standard deviation (SD) or median with the corresponding first and third quartiles (Q1–Q3)

**Table 2:** Burn injury mechanism, place of burn and cause of deaths in survivors and non-survivors in a Malaysian burns intensive care unit

Variable	All episodes, *n* (%)	Survivor, *n* (%)	Non-survivor, *n* (%)
	(*n* = 393)	(*n* = 345)	(*n* = 48)
Burn injury mechanism			
Thermal	343 (87.3)	303 (87.8)	40 (83.3)
Chemical	31 (7.9)	25 (7.2)	6 (12.5)
Electrical	15 (38.5)	14 (4.1)	1 (2.1)
Others	4 (1.0)	3 (0.9)	1 (2.1)
Place of burn			
Household	189 (48.1)	168 (48.7)	21 (43.8)
Industrial	137 (34.9)	121 (35.1)	16 (33.3)
Road traffic accident	29 (7.4)	25 (7.2)	4 (8.3)
Non-accidental	43 (10.9)	33 (9.6)	10 (20.8)
Others	87 (22.1)	82 (23.8)	5 (10.4)
Cause of death			
Sepsis	–	–	19 (39.6)
Inhalation injury	–	–	16 (33.3)
Multi-organ failure	–	–	9 (18.8)
ARDS	–	–	3 (6.3)
Others	–	–	1 (2.1)

*ARDS* acute respiratory disdress syndrome

### Odds ratio and logistic regression

Risk factors of interest were analyzed using simple logistic regression. Risk factors of TBSA > 20%, degrees of burn, inhalation injury and early SIRS were all significant predictors of mortality (Table 3). Patients with TBSA greater than 20% had 16 times greater risk of death with a *p* value of 0.037. Inhalation injuries had the most significant impact of mortality with 32 times risk of death with *p* < 0.001. All identified predictors of death were universal in other studies apart from early SIRS which is a significant predictor of death in this study. Early SIRS on admission was also associated with 13 times risk of death with *p* < 0.001.

**Table 3:** Logistic regression for predictors associated with mortality in severe burns in a Malaysian burns intensive care unit

Variable	Odds ratio	95% CI		*p* value
		Lower	Upper	
Age group (years)				
≤ 18	1.00 (reference)	–	–	–
19–49	2.08	0.08	0.48	0.331
50–64	3.20	0.64	16.13	0.159
65–74	5.82	0.93	36.28	0.059
≥ 75	2.00	0.16	24.92	0.590
Gender				
Male	1.08	0.54	2.16	0.839
Female	1.00 (reference)	–	–	–
Place of burn				
Household	0.82	0.45	1.51	0.521
Industrial	0.93	0.49	1.76	0.813
Road traffic accident	1.16	0.39	3.50	0.787
Non-accidental	2.49	1.14	5.45	0.023
Others	0.37	0.14	0.97	0.044
% TBSA				
0 – 10%	1.00 (reference)	–	–	–
10 – 20%	1.16	0.25	5.29	0.849
20 – 30%	4.24	1.09	16.44	0.037
30 – 40%	11.47	2.81	46.84	0.001
≥ 40%	48.36	15.72	148.76	< 0.001
Degree of burn				
Partial-thickness	0.38	0.18	0.80	0.010
Full-thickness	3.12	1.54	6.32	0.002
Inhalation injury	15.66	7.56	32.42	< 0.001
SIRS at presentation	6.21	2.99	12.88	< 0.001
LOS (days)				
0 – 10	1.00 (reference)	–	–	–
10 – 20	1.56	0.74	3.28	0.240
20 – 30	1.12	0.40	3.16	0.826
30 – 40	1.20	0.33	4.33	0.787
≥ 40	1.95	0.72	5.26	0.189

*CI* confidence interval, *% TBSA* percentage of total body surface area, *SIRS* systemic inflammatory response syndrome, *LOS* length of stay

### Survival analysis using risk factors of interest

**Fig. 1:**
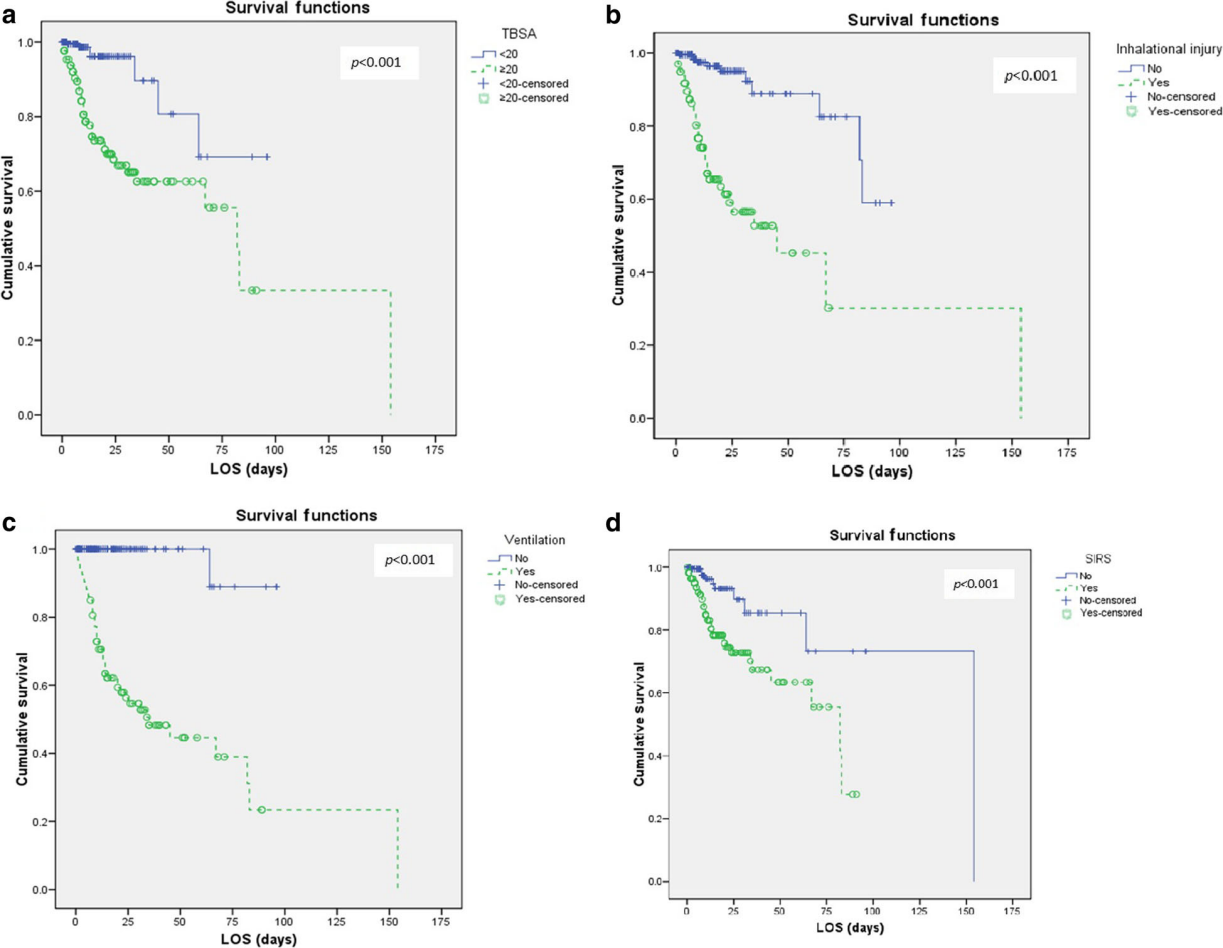
Kaplen-Meier survival curve of significant predictors of burn mortality. **a** Total body surface area (TBSA)>20%. **b** Inhalational injury. **c** Mechanical ventilation. **d** Early systemic inflammatory response syndrome (SIRS)

Survival analysis with log-rank test was performed and found that TBSA > 20% (*p* < 0.001), early SIRS (*p* < 0.001), ventilated patients (*p* < 0.001) and inhalation injury (*p* < 0.001) were associated with poorer survival outcome (Fig. 1). These variables showed an increased probability of death after 75 days of admission. The results are invariably noticeable in patients with inhalation injury and ventilated patients where the survival probability reduces to less than 20% after 50 days of hospital stay.

## Discussion

Older age, a larger burn TBSA and inhalation injury are well-known predictors of burn mortality [11–13]. The result of our study is similar, which shows that the presence of inhalation injury and large TBSA burn is predictive of burn mortality. From our study, older age had a close to significant *p* value of 0.056 and odds ratio of 5 for burn mortality which showed a strong correlation with death. The main reason for the close to significant *p* value was probably due to the small sample population of elderly patients in our burn registry. Despite that, older age is a known mortality predictor of burn injury due to a decrease in immune function with physiological thinning of skin [14]. This contributes to more than a quarter of burn mortality in the entire burn population across all ages as reported by Santos et al. [6]. Furthermore, the incidence of co-morbidities of hypertension, diabetes and pulmonary diseases rises within the ageing population. Co-morbidities such as chronic obstructive pulmonary disease are a contributing factor leading to death in older patients [6]. Due to physiological factors of a weakened immune system, elderly burn-injured patients are likely to succumb to death from infective complications during hospitalizations or during the resuscitative phase [15, 16]. In addition, the moist and devitalized burn tissue provides a suitable medium for proliferation of bacteria [17, 18]. Common bacterial isolate from blood cultures due to burned wound infection is *Pseudomonas aeruginosa* with a prevalence rate of 8.4% [7].

The overall mortality rate in this current study is 12.2% which is considered acceptable from a burn centre from a developing nation. When compared to another developing nations, burn centre of India reported that the mortality rate goes up to 60.8% [19]. In contrast to neighbouring Southeast Asia and developed country of Singapore, a lower mortality rate of 4.6% was reported in 2003 [20]. This data must be viewed with caution as the admission criteria might differ between centres which lead to a variation of the death rates. Due to better facilities and more bed availability, patients least serious burn injuries were also admitted and included in the study from Singapore which may account for the low mortality rate. Nevertheless, it gives us an estimation and comparison of our capability in terms of death rates in managing severe burn patients. The burn admission criteria and referral criteria in our centre are identical. However, due to a limitation in bed availability, not all burn cases which fit the criteria of burn admission were admitted. This may lead to the large discrepancy in mortality rate as reported by Ganesemoni et al. of burn patients in India [19]. The sample population of this study had a majority of patients with severely critical burn, and due to bed limitation, burn injury patients that fit the admission criteria were treated at other non-burns centres [3]. Naturally, if only severe cases were included, mortality rate goes up and the rate drops when least severe cases were admitted and analyzed together.

Burn injuries in the paediatric population are the other end of the extreme age with poor outcomes even in burn centres of developed nations. This is due to the formation of contractures and disfigurement which may lead to psychological effects in early adulthood [21]. It is interesting to note that if a paediatric burn occurred in a developing nation of Africa, the mortality rate is three times higher compared to children worldwide. In the burn injury trends observed mainly in the 2-year-old with a 30-day mortality rate of 7% as reported by Chelidze et al., majority of injuries were unintentional with domestic scalding burns of 98% in a paediatric burn population in Africa [22]. Our study did not show genders and age as significant predictors as reported in multiple studies. Reasons for deaths in the paediatric burn injuries include the physiologic thinning of the skin, poorer immune function and reduction in strength for post-treatment physiotherapy and rehabilitation [15, 20, 23]. Our sub-analysis age groups revealed that the majority of deaths occurred in 19–49 years of age which is the active working class Malaysian population. The analysis of age subgroups had only 6% of patients in the geriatric age population of 65 years and above. In contrast to the developed nations, the number of geriatric population rises steadily with improving medical health care access [24]. Gender was a predictor in another study which can be due to socio-cultural differences [13]. In India, burn injuries were more common in the female gender by acid or flame burn attacks [25]. The events leading to these injuries were related to suicide and homicide that tend to be more severe [26]. This shows a wide variation in burn injuries and death which is contributed due to the access to health care services, socio-economic differences and cultural with gender variation from other nations.

Burn injuries over 20% TBSA and inhalation injury are significant predictors in this current study which were similar to other reports [27, 28]. SIRS on an early presentation from admission may be used as a predictor of mortality as it was a significant predictor for death and survival (Fig. 1). A study in an American burns unit revealed that SIRS was not sensitive enough to predict sepsis in burn patients [29]. This has led us to suggest that SIRS is more commonly due to the severity of burn injury itself and leads to the overwhelming immune response to injury rather than infection. This can be explained by the previous report on the observation of rising interleukin (IL) in burn injury patients [30, 31]. The level of IL-6 is positively correlated with the severity of burn [30]. Another study in Taiwan demonstrated specific increment of IL-6 and IL-10 levels within the non-survivors burned patients [31]. The levels of IL-6 have a role in predicting the severity of injury in burned patients from the previous studies [32]. However, to generalize its use in usual practice especially in countries with a limited resource is cost ineffective. Therefore, we recommend the SIRS criteria as one of the important key predictors for burn patient mortality. This allows more detailed triaging of burn patients for intensive care bed allocation. By identifying burn patients at high risk of mortality, it is useful for the allocation of resources during a disaster which many will require multiple infusion pumps, ventilator and inotropic agents.

### Limitations

We acknowledge that the data is from a single-centre burn unit experience, and our numbers may pale in comparison with other published studies as multicentre studies are required to garner a large number of patients. Likewise, in comparison with similar single-centre prospective published study, the results were comparable [1, 2]. However, the authors believe that this is the current situation being reflected in other centres in Malaysia managing burn injuries. This data is important as we found no published local Malaysian data on other variables of burn mortality predictors.

### Strength

The data collected includes all burn patients which reduces the sampling bias. From our study, we identified early SIRS to be the third most important prognostic factor in mortality besides TBSA and inhalation injury. In addition, this study gives an estimate of our regional burn outcome managed by a single burns surgeon.

## Conclusion

From this study which was conducted in a Malaysian BICU, the predictors of mortality identified were TBSA > 20%, early SIRS, mechanical ventilation and inhalation injury which were associated with poorer survival outcome. Early SIRS and mechanical ventilation were significant predictors of mortality. The immunological response differs from individual patients and influenced by the severity of the burn. SIRS on admission is an important predictor of death and may represent the severity of the burn injury. It is an inexpensive way of measuring the immunological response indirectly in comparison with costly laboratory and biochemical test specific for immunological markers of burn injuries. Patients who required mechanical ventilation were associated with mortality. This relates to the severity of associated lung injury sustained by individual patients due to the burn. Early SIRS and ventilated patients were novel predictors of death identified in this study. The use of these two predictors in addition to the universal well-known predictors of burn mortality (inhalation injury and TBSA burn) gives added variables for outcome prognostication and mortality risk counselling in severely burned patients. Furthermore, this study is important in Malaysia to identify the mortality risk factors of burn which may be used for local clinicians to stratify risk and predict outcome comparison for burn injuries.

## Abbreviations

BICU: Burns intensive care unit
HSI: Hospital Sultan Ismail
IL-10: Interleukin-10
IL-6: Interleukin-6
SIRS: Systemic inflammatory response syndrome
TBSA: Total body surface area
